# Comparison of chitosan nanoparticles containing *Lippia citriodora* essential oil and citral on the induction of apoptosis in A375 melanoma cells

**DOI:** 10.1186/s12906-023-04268-2

**Published:** 2023-12-01

**Authors:** Abolfazl Karimivaselabadi, Mahmoud Osanloo, Ali Ghanbariasad, Elham Zarenezhad, Hossein Hosseini

**Affiliations:** 1 Department of Clinical Biochemistry, Faculty of Medicine, Mashhad University of Medical Sciences, Mashhad, Iran; 2 Department of Medical Nanotechnology, School of Advanced Technologies in Medicine, Fasa University of Medical Sciences, Fasa, Iran; 3 Department of Medical Biotechnology, School of Advanced Technologies in Medicine, Fasa University of Medical Sciences, Fasa, Iran; 4 Noncommunicable Disease Research Center, Fasa University of Medical Sciences, Fasa, Iran

**Keywords:** Melanoma, Essential oils, Citral, L. citriodora, Chitosan, Nanoparticles

## Abstract

**Background:**

Using nanoparticles containing L. citriodora EO and citral has shown potential in treating skin disorders such as melanoma.

**Methods:**

In this study, GC‒MS was used to analyze the chemical composition of L. citriodora essential oil (EO). The ion gelation method prepared free chitosan nanoparticles and chitosan nanoparticles containing L. citriodora EO and citral. The successful loading of the EO and citral was evaluated using ATR-FTIR. The DPPH assay measured the antioxidant effect of citral, L. citriodora EO, Citral-ChiNPs, L. citriodora-ChiNPs, and Free-ChiNPs. A375 melanoma cell viability was assessed using the MTT assay. The qPCR technique was employed to evaluate the expression of apoptotic genes, and flow cytometry was used to detect apoptosis.

**Results:**

This study showed that in equal concentrations, the antioxidant properties of chitosan nanoparticles containing citral were greater than those of chitosan nanoparticles containing L. citriodora. The IC50 values of chitosan nanoparticles containing citral, L. citriodora EO, and their nonformulated states were 105.6, 199.9, 136.9, and 240 µg/ml, respectively. The gene expression results showed that the ratio of the expression of the apoptosis gene to the inhibitory gene was higher than 1 in all the samples, indicating that the conditions for apoptosis were present. Flow cytometry confirmed cell apoptosis, with 93.5 ± 0.3% in chitosan nanoparticles containing citral, 80 ± 0.2% in chitosan nanoparticles containing L. citriodora EO, 63 ± 0.3 in citral, and 42.03% in L. citriodora EO-treated cells.

**Conclusion:**

The results showed that using the Nano form of L. citriodora and citral increased their efficiency in apoptosis pathways and their toxicity against 375 melanoma cancer cells.

## Background

International Agency for Research on Cancer; 325,000 new skin cancer cases and 57,000 deaths were recorded in 2020. In addition, it is estimated that between 2020 and 2040, the number of newly diagnosed cases will exceed 500,000 per year, and the number of fatalities will surpass 100,000 per year [1]. There are two primary skin cancer types: nonmelanoma and malignant melanoma. Cutaneous melanoma is a cancerous growth in melanocytes. These cells are located in the basal layer of the epidermis and are responsible for producing melanin, which determines skin color [2]. Malignant melanoma is the most lethal form of skin cancer, posing a significant health challenge due to its resistance to radiation therapy, chemotherapy, and immunotherapy [3–5]. Investigation of 42 melanoma cell lines at the transcriptome level has shown that the A-375 cell line has minor sensitivity to chemotherapy [6].

Essential oils (EOs) are an excellent resource for developing new anticancer agents due to various compounds with anticancer, antitumor, and antiproliferative properties and few side effects [7, 8]. In addition, they also have a high potential for antibacterial, antiviral, and antioxidant effects [9, 10]. However, as their low solubility in water should be formulated, preparing nanostructures containing EOs is a promising approach for solubility and efficacy improvement [11, 12]. Chitosan nanoparticles with biodegradability, biocompatibility, and mucosal adhesion properties are among the most common carriers [13, 14]. In addition, they have a variety of beneficial properties, including antimicrobial, anti-inflammatory, antioxidant, anticoagulant, antitumor, antihypertensive, hypocholesterolemic, and antidiabetic effects [15].

*Lippia citriodora* (Lemon verbena) has been used in traditional medicine to relieve various symptoms, such as digestive issues, fever, cold, asthma, diabetes, spasms, insomnia, and anxiety [16]. In addition, research has shown that the flavonoid and phenol content in *L. citriodora* EO contributes to its significant antioxidant activity [17, 18]. Moreover, some studies have demonstrated the free radical scavenging abilities of *L. citriodora* to infusion against superoxide, hydroxyl radicals, and hypochlorous acid in vitro, in vivo, and ex vivo systems [19]. Interestingly, some researchers have suggested that *L. citriodora* EO can inhibit the proliferation of cancer cells [20, 21]. Furthermore, citral is a significant component of EOs obtained from various lemon-scented herbal plants, such as *L. citriodora* EO. Citral exhibits biological effects, such as anticancer, antioxidant, and antibacterial activities [22, 23]. Moreover, citral possesses antitumor properties in mouse cancer models when administered orally as part of their diet [24, 25].

The present study aimed to evaluate and compare the cytotoxicity and antioxidant properties of L. citriodora EO, citral, and chitosan nanoparticles containing each against A375 human melanoma cells. After that, qPCR and flow cytometry techniques were used to measure their effects on apoptosis regulator genes and the percentage of apoptotic cells, respectively.

## Materials and methods

### Materials

The Pasteur Institute of Iran supplied the A-375 melanoma cell line. MTT powder (3-(4,5-dimethylthiazol-2-yl)-2,5-diphenyl tetrazolium bromide), citral, tripolyphosphate (TPP) and DPPH powder (1,1-diphenyl-2picrylhydrazyl) were purchased from Sigma Aldrich (USA). Chitosan’s low molecular weight, PBS tablet (phosphate buffered saline), and Tween 20 were purchased from Merck (Germany*). L. citriodora* EO was purchased by Giah Essence Phytopharm Co. (Iran). RPMI 1640 culture medium, trypsin, penicillin-streptomycin, and FBS were obtained from Shellmax (China). cDNA synthesis kits and TRIzol were purchased from Yektatajhiz (Iran). SYBR green Master Mix High ROX was purchased from Ampliqon (Denmark), and the Annexin-V/FITC apoptosis detection kit was purchased from Mab Tag (Germany).

### GC-MC analysis

*L. citriodora* EO was analyzed by an Agilent 6890 gas chromatography device (USA). First, the sample was diluted with n-hexane, and one microliter was injected into the BPX5-type column of this device with a length of 30 m, an inner diameter of 0.25 mm, and a layer thickness of 0.25 micrometers. The temperature program of the column was such that at first, the oven was stopped at 50 °C for five minutes, then it was increased with a thermal gradient of three degrees per minute until it reached 240 °C. Then, the temperature gradient was increased to 15 °C per minute until the temperature reached 300 °C and was stopped at this temperature for three minutes. The injection chamber was split from one to 35, and its temperature was 250 °C. The carrier gas of the injection chamber was helium at a rate of 0.5 mm per minute. An Agilent 5973 mass spectrometer with an ionization voltage of 70 electron volts, an ionization source temperature of 220 °C, and an EI ionization method were used in this device. The detectors used to scan the masses were set in the range of 40 to 500. CHEMSTATION software was used for the identification of compounds.

### Preparation of nanoparticles

The ionic gelation method was used to prepare chitosan nanoparticles [26]. For this purpose, first, *L. citriodora* EO (0.16% v/v) and citral (0.16% v/v) were mixed separately with Tween 20 (0.24% v/v) for 4 min at 2000 rpm. Then, 4500 µl of chitosan solution (0.2% w/v chitosan dissolved in 1% acetic acid) was added dropwise to each sample and stirred for 45 min at room temperature at 2000 rpm. Then, 500 µl of TPP aqueous solution (1- 0.2% w/v) was added dropwise and stirred at room temperature for 60 min at 2000 rpm to obtain chitosan nanoparticles containing *L. citriodora* EO (L. citriodora-ChiNPs) and chitosan nanoparticles containing citral (Citral-ChiNPs). The above steps were also performed to make chitosan without anything (Free-ChiNPs).

The sizes of Free-ChiNPs, Citral-ChiNPs, and L. citriodora-ChiNPs were investigated using a DLS-type apparatus (K-One nano Ltd, Korea). To calculate particle size distributions (SPAN), D90—D10 ⁄ D50 was used. D is the diameter, and D10, D50, and D90 are percentiles of particles with a diameter lower than these values. In addition, the zeta potential of the nanoparticles was investigated using a DLS HORIBA SZ-100 (Japan) with the following settings: dispersion medium viscosity: 0.891 mPa·s, temperature of the holder: 25.2 °C, electrode voltage: 3.3 V and conductivity: 0.574 mS/cm. Moreover, to determine the morphology of Free-ChiNPs, Citral-ChiNPs, and L. citriodora-ChiNPs, a transmission electron microscope (TEM) with 100 kv accelerating voltage was used (Philips EM208S, Netherlands).

### Confirmation of loading of *L. citriodora* EO and citral by chitosan nanoparticles

To investigate the chemical composition of nanoparticles and to prove the loading of *L. citriodora* EO and citral in chitosan nanoparticles. ATR-FTIR spectra of citral, *L. citriodora* EO, Free-ChiNPs, Citral-ChiNPs, and L. citriodora-ChiNPs were recorded in the range of 400–4000 cm^− 1^ using a spectrometer machine (Brooker, Tensor II, USA).

### DPPH assay

The DPPH assay measured the antioxidant effect of citral, *L. citriodora* EO, Citral-ChiNPs, L. citriodora-ChiNPs, and Free-ChiNPs at 25–800 µg/ml concentrations. Ethanol (96%) was used to prepare a serial dilution of each sample. First, 50 µl of each sample and 50 µl of DPPH solution (0.3 mM) were poured into each well. In addition, six wells containing 50 µl of ethanol and 50 µl of DPPH solution were considered controls. Next, the plate was incubated away from light for 30 min to complete the reaction, and finally, the absorbance of the wells was read by a plate reader (Synergy HTX Multi-Mode Reader, USA) at a wavelength of 517 nm. Finally, the percentage of DPPH scavenging activity at each concentration was calculated by ([OD Control-OD Sample]/OD Control ×100).

### MTT assay

A375 cells were cultured in RPMI 1640 complete medium containing 10% fetal bovine serum and 1% penicillin/streptomycin. The cells were grown at 37 °C and 5% CO2. Then, the cells were seeded in 96-well plates (10,000 cells per well) and incubated for 24 h. Next, the culture medium of each well was replaced with 50 µl of RPMI complete culture medium. After that, the cells were treated with citral, *L. citriodora* EO, Citral-ChiNPs, L. citriodora-ChiNPs, and Free-ChiNPs at concentrations of 25, 50, 100, 200, 400, and 800 µg/ml and incubated for 24 h.

After 24 h, 100 µl of MTT solution (0.5 mg/ml in PBS) was added to the wells and then incubated for 4 h at 37 °C. Then, 100 µl of DMSO was added to each well and shaken for 5 min, and the produced purple crystals were dissolved. After that, the absorbance of each sample and control (untreated with sample) well was read using an ELISA reader (Synergy HTX Multi-Mode reader, USA) at a wavelength of 570 nm. Finally, the cell viability at each concentration was calculated by (OD sample/OD control) ×100.

### qPCR technique

The qPCR technique was employed to evaluate the expression of apoptotic-involved genes, including Bax pro-apoptotic and Bcl-2 anti-apoptotic genes. Initially, 50,000 A375 cells per well were seeded in 6-well plates. The cells were then treated with Free-ChiNPs (200 µg/mL), *L. citriodora*-ChiNPs (200 µg/mL), *L. citriodora* EO (200 µg/mL), citral (100 µg/mL), and Citral-ChiNPs (100 µg/mL) followed by incubation for 24 h at 37 °C. Next, total RNA was extracted using the TRIzol RNA extraction kit (Yektatajhiz). The wells were washed with PBS, and the liquid content was discarded.

The cells were centrifuged for 10 min at 700 g, and then 500 µl of TRIzol was added to the pellet and shaken for 6 min at room temperature. Then, 100 µl of chloroform was added, and after staying at room temperature for 3 min, the sample was centrifuged for 10 min at 15,000 g. Next, 500 µl of isopropanol was added to the supernatant in a new microtube, kept at -20 °C for 10 min, and then centrifuged for 10 min at 15,000 g. Next, 200 µl of 75% alcohol was added to the residue and centrifuged for 5 min at 15,000 g; the ethanol was discarded, and the obtained pellets were dried at 50 °C (two times). After that, the extracted RNA was dispersed in DEPC water and assessed for quality and quantity using a Nanodrop apparatus (Synergy HTX Multi-Mode Reader, USA). The purity of RNA and protein contamination was determined by measuring the absorbance (OD) ratio at 260 and 280 nm. A ratio of > 1.8 was considered indicative of a pure sample.

A cDNA synthesis kit (Yektatajhiz, Iran) was used to synthesize cDNA. Initially, the extracted total RNAs were mixed with oligo dT and DEPC water and incubated for 5 and 1 min at 70 °C and 4 °C, respectively. Subsequently, 5X strand buffer, dNTPs (10 mM), RNasin (40 U/µL), and M-MLV were added to the mixture and subjected to the Bio-Rad Thermocycler apparatus. The thermal program was set at 60 min at 42 °C, and the resulting cDNAs were stored at − 20 °C. Amplification was performed using a qPCR machine (StepOnePlus, Applied Biosystems, USA) and RealQ Plus 2x Master Mix Green high ROX™ (Amplicon, Denmark). The master mix (Green High Rox), forward and reverse primers for each gene (Pishgam Biotech Co., Tehran, Iran, see Table 1), and cDNA template were combined to a final volume of 20 µl using DEPC water. Amplification reactions were then carried out under the following conditions: 10 min at 95 °C, 40 cycles of 95 °C for 15 s, 55 °C for 30 s, and 72 °C for 30 s. The relative fold changes in the expression of target genes (Bax and Bcl-2) with β-actin as an internal control were normalized using the 2^˗ΔΔCT^ method, where ΔCT = CT target ˗ CT reference, ΔΔCT = ΔCT test sample ˗ ΔCT control sample, and relative expression = 2^˗ΔΔCT^.

**Table 1:** Primer sequences of genes

Gene name	Primer sequence
β-actin	Forward: 5′ - TCCTCCTGAGCGCAAGTAC − 3′ Reverse: 5′ - CCTGCTTGCTGATCCACATCT − 3′
Bax	Forward: 5′ - CCCGAGAGGTCTTTTTCCGAG − 3′ Reverse: 5′ - CCAGCCCATGATGGTTCTGAT − 3′
Bcl-2	Forward: 5′ - GGTGGGGTCATGTGTGTGG − 3′ Reverse: 5′ - CGGTTCAGGTACTCAGTCATCC − 3′

### Apoptosis detection using flow cytometry

To confirm the induction of apoptosis, A375 cells were seeded in 6-well plates and treated with different formulations of Free-ChiNPs (200 µg/mL), *L. citriodora*-ChiNPs (200 µg/mL), *L. citriodora* EO (200 µg/mL), citral (100 µg/mL), and Citral-ChiNPs (100 µg/mL) for 24 h at 37 °C. The Annexin-V/FITC/PI Apoptosis Detection Kit protocol (MabTag, GmbH, Germany) confirmed apoptosis. The cells were harvested, washed with PBS, and resuspended in 1x Annexin-V binding buffer. Then, Annexin-V conjugate and propidium iodide solution were added to the cell suspension and incubated for 20 min in the dark at room temperature. After incubation, 1x binding buffer was added, mixed gently, and analyzed using flow cytometry (BD FACSCalibur, USA). FlowJo software (BD, Becton, Dickinson, and Company) was used to determine the numbers of viable cells, cells undergoing necrosis (positive for PI), early apoptosis (positive for Annexin-V/FITC), and late apoptosis (double-positive for Annexin-V/FITC and PI).

## Results

### *L. Citriodora* EO analysis by GC‒MS

The compounds identified in *L. citriodora* EO using GC‒MS are presented in Table 2. The five most abundant compounds found in the EO were citral (25.97%), limonene (12.09%), curcumin (10.27%), caryophyllene (6.91%), and 1,8-cineole (5.42%).

**Table 2:** Identified compounds in *L. citriodora* EO using GC‒MS analysis

NO.	Compound	%	Retention time	Kovats Index	Type
1	α-Pinene	0.58	11.31	939	MH^1^
2	Sabinene	1.48	13.37	975	MH
3	Octen-3-ol	0.45	13.94	979	MH
4	Hepten-2-one(6-methyl-5)	3.36	14.22	986	MH
5	Octanol(3)	0.18	14.70	991	MH
6	Limonene	12.09	16.35	1029	MH
7	1,8-Cineole	5.42	16.53	1031	MH
8	Ocimene(z-beta)	0.14	16.68	1037	MH
9	Ocimene(E-beta)	2.20	17.22	1050	MH
10	γ -Terpinene	0.10	17.88	1060	MH
11	Terpinolene	0.64	20.09	1089	MH
12	Trans-chrysanthemal	0.62	22.71	1153	Other
13	Terpinen-4-ol	0.82	24.27	1177	MO^2^
14	α-Terpineol	1.31	25.06	1189	MO
15	Nerol	0.73	26.30	1230	MO
16	Citral	25.97	28.52	1267	MO
17	α-Copaene	0.83	33.03	1377	SH^3^
18	Geranyl acetate	0.82	33.21	1381	SH
19	β-bourbonene	0.59	33.40	1388	SH
20	Caryophyllene(E)	6.91	35.00	1419	SH
21	α-Humulene	0.44	36.55	1455	SH
22	Aromadendrene(allo-)	0.71	36.73	1458	SH
23	trans Cadina-1(6),4-diene	0.48	37.34	1477	MO
24	Curcumene(ar)	10.27	37.58	1481	SH
25	γ -Curcumene	2.02	38.09	1483	SH
26	Bicycloelemene	4.17	38.23	1500	SH
27	γ -Patchoulene	2.60	38.69	1502	SH
28	Cadinene	1.29	39.9	1539	SH
29	Nerolidol(e)	1.07	40.74	1563	SO^4^
30	Spathulenol	3.24	41.65	1578	SO
31	Caryophyllene oxide	2.17	41.83	1583	SO
32	α-Cadinol	0.53	44.67	1654	SO

^1^Monoterpene hydrocarbons, ^2^Oxygenated monoterpenes, ^3^Sesquiterpene hydrocarbons, ^4^Oxyganated sesquiterpenes

### Physicochemical properties of the prepared nanoparticles

**Fig. 1:**
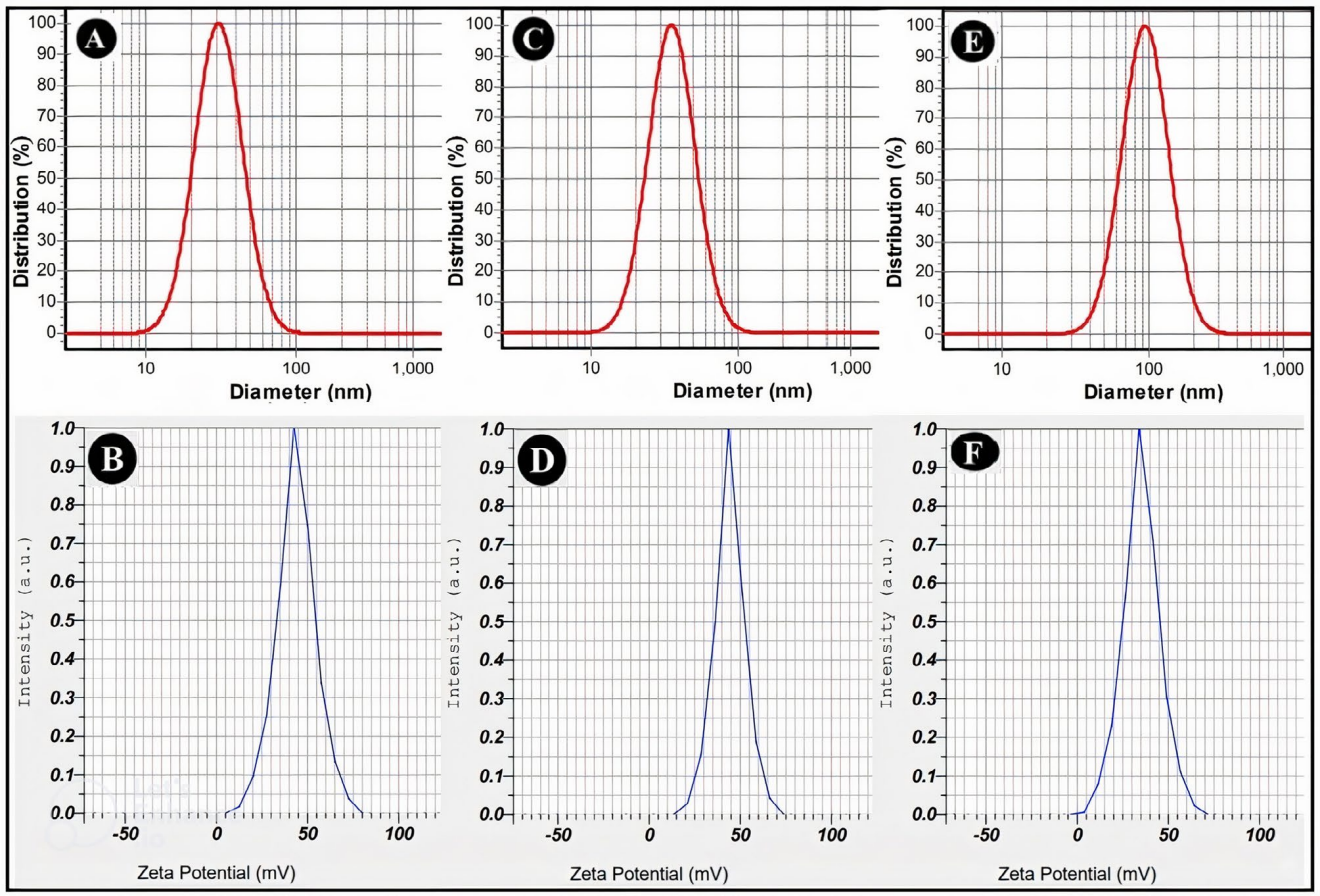
DLS and Zeta potential diagrams of Free-ChiNPs (**A** and **B**), Citral-ChiNPs (**C** and **D**), and L. citriodora-ChiNPs (**E** and **F**)

**Fig. 2:**
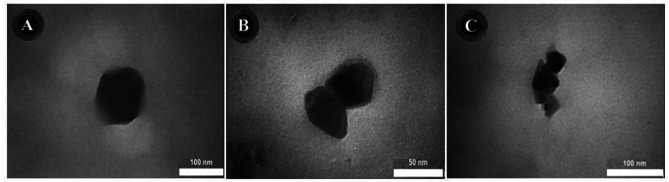
TEM images of **A**: Free-ChiNPs, **B**: Citral-ChiNPs, and **C**: L. citriodora-ChiNPs

Fifteen nanoformulations were prepared to obtain chitosan nanoparticles with desired properties, i.e., droplet size < 200 nm and SPAN < 1. Table 3 shows that different TPP concentrations were screened; samples containing 0.2% TPP [13–15] showed the best size characteristics. Their DLS and zeta potential diagrams are shown in Fig. 1. The particle size, SPAN value, and zeta potential of free-ChiNPs were 31 ± 5 nm, 0.97, and 43 ± 1 mV, respectively. In addition, these values for Citral-ChiNPs were 35 ± 4 nm, 0.94, and 44 ± 1 mV, and for L. citriodora-ChiNPs, they were 94 ± 6 nm, 0.98, and 35 ± 3 mV. Moreover, their TEM images are depicted in Fig. 2.

**Table 3:** Ingredients of the prepared nanoparticles and their size analyses

Sample	Chitosan (%)	TPP^1^ (%)	*L. citriodora* EO	Citral	tween 20 (%)	Particle size (nm)	SPAN^2^
1	0.2	0.1	0.16	_	0.24	403	0.98
2	0.2	0.1	_	0.16	0.24	267	0.96
3	0.2	0.1	_	_	0.24	245	0.99
4	0.2	0.8	0.16	_	0.24	391	3.5
5	0.2	0.8	_	0.16	0.24	275	0.97
6	0.2	0.8	_	_	0.24	238	1.2
7	0.2	0.6	0.16	_	0.24	667	1.4
8	0.2	0.6	_	0.16	0.24	708	1.4
9	0.2	0.6	_	_	0.24	532	0.99
10	0.2	0.4	0.16	_	0.24	258	1.7
11	0.2	0.4	_	0.16	0.24	202	0.99
12	0.2	0.4	_	_	0.24	146	0.98
13	0.2	0.2	0.16	_	0.24	94	0.98
14	0.2	0.2	_	0.16	0.24	35	0.94
15	0.2	0.2	_	_	0.24	30	0.97

^1^Tripolyphosphate. ^2^Particle size distribution (value < 1 has a narrow distribution)

**Fig. 3:**
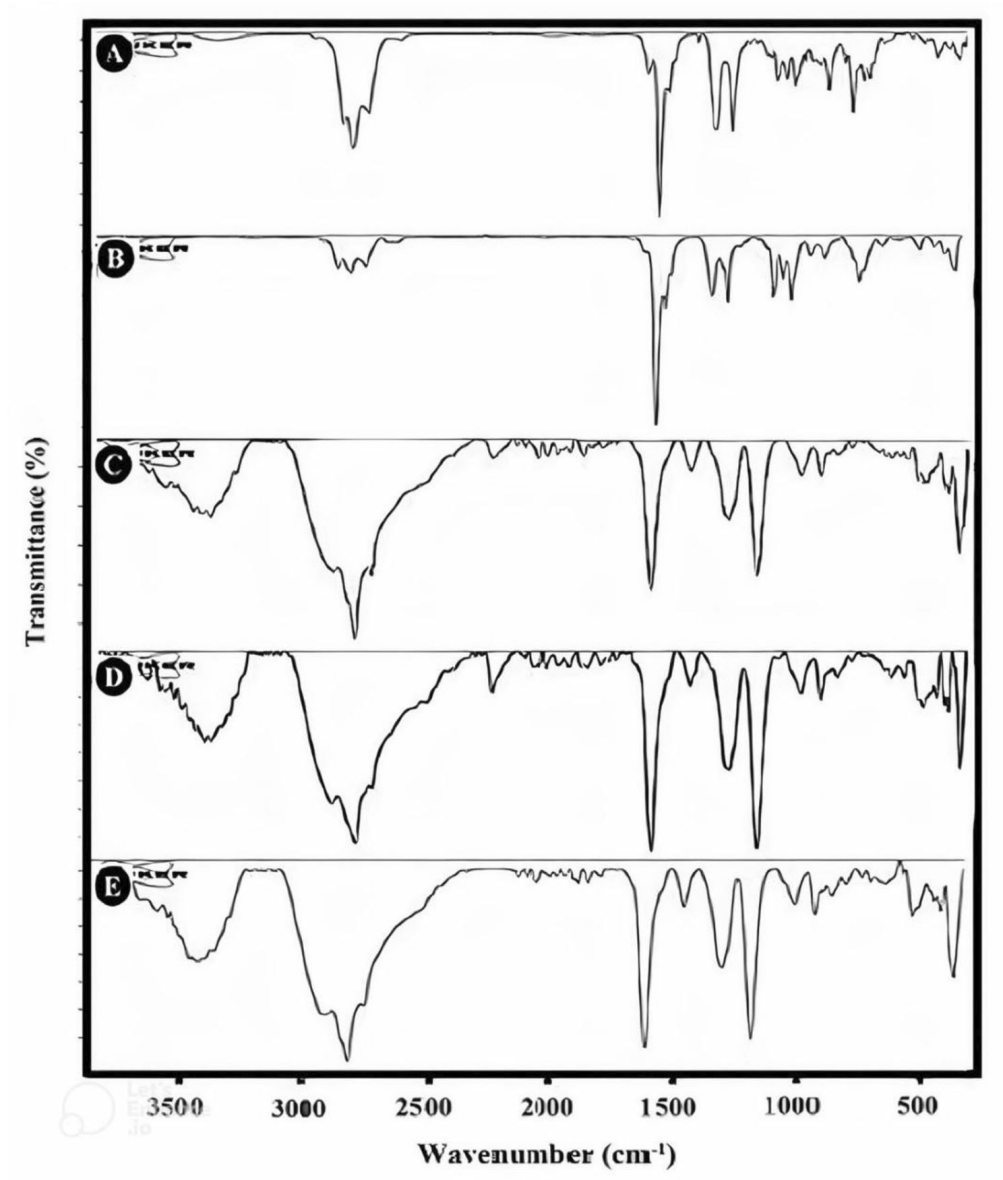
ATR-FTIR spectra of **A**: *L. citriodora* EO, **B**: citral, **C**: free-ChiNPs, **D**: citral-ChiNPs, and **E**: L. citriodora-ChiNPs

The ATR-FTIR spectra of *L. citriodora* EO (Fig. 3A) displayed a broad peak at 3469 cm^− 1,^ which can be attributed to the stretching vibration of the hydroxyl group due to hydrogen bonding in phenolic and alcoholic compounds in EO. The bands at 2961, 2922, and 2857 cm^− 1^ can be attributed to the stretching vibration of CH, and the strong peak at 1675 cm^− 1^ can be attributed to the stretching vibration of carbonyl groups. The 1633 and 1445 cm-1 bands can be related to C = C stretching vibrations.

The ATR-FTIR spectra of citral (Fig. 3B) showed that the band at 3009 cm^− 1^ can be related to the stretching vibration of CH in sp2 groups due to alkene structures. The bands at 2967, 2915, and 2856 cm^− 1^ can be attributed to the stretching vibration of CH in sp3 groups; the band at 1671 cm^− 1^ can be related to the stretching vibration of carbonyl groups. The 1632 and 1441 cm-1 bands can be related to unsaturated C = C in citral.

The ATR-FTIR spectrum of free-ChiNPs (Fig. 3C) displayed a broad band at approximately 3200–3600 cm^− 1,^ which can be related to the hydroxyl group due to hydrogen bonding. The band at approximately 3009 cm^− 1^ is related to = CH, and the peaks at approximately 2925 and 2855 cm^− 1^ can be related to –C-H starching vibration. The sharp peak at 1711 cm^− 1^ can be related to C = O stretching in Tween and chitosan. Two peaks at 1278 and 1098 cm^− 1^ belong to anti-symmetric stretching vibrations of PO2 groups in TPP (TPP) ions. These bands demonstrated the ionic links between the NH3 + of chitosan and the anionic structure of TPP.

The ATR-FTIR spectra of Citral-ChiNPs (Fig. 3D) displayed a broad peak at approximately 3200–3700 cm^− 1^ related to OH groups due to hydrogen bonding between citral, chitosan, and TPP. The band at 3015 cm^− 1^ can be related to the = C-H stretching vibration. The 2921 and 2854 cm-1 peaks can be related to –C-H stretching vibrations. The bands at 2402 and 2360 cm^− 1^ can be attributed to CO2. The strong and characteristic band at 1711 cm^− 1^ is related to a carbonyl group. Two characteristic bands were displayed at 1550 cm^− 1^ (C-O-C) and 1278 cm^− 1^ (amide II), which can be related to the complex preparation with electrostatic linking between the NH3 + structure of chitosan and phosphoric groups of TPP between the ChiNps. All the other peaks appear in the spectrum of citral at the same wavenumber; it could be confirmed that citral was encapsulated into ChiNPs.

The ATR-FTIR spectrum of L. citriodora-ChiNPs (Fig. 3E) showed that the broad peak between 3200 and 3600 cm^− 1^ corresponded to OH groups due to hydrogen bonding between EO, chitosan, and TPP. The peaks at 3011 cm^− 1^ can be allocated to the = C-H stretching vibration. The 2926 and 2857 cm-1 peaks can be attributed to -C-H stretching vibrations. The strong and characteristic band at 1711 cm^− 1^ was related to the carbonyl group in chitosan, EO. A characteristic peak at 1279 cm^− 1^ related to C-N stretching demonstrated the complex preparation with linking electrostatic between NH3 + groups of chitosan and anionic groups of TPP. The band at 1551 cm^− 1^ can be allocated to C-N stretching and relates to the amide structure. All the other bands appear in the spectrum of EO at the same wavenumber; it could be confirmed that the EO was encapsulated into ChiNPs.

### Comparison of antioxidant effects

**Fig. 4:**
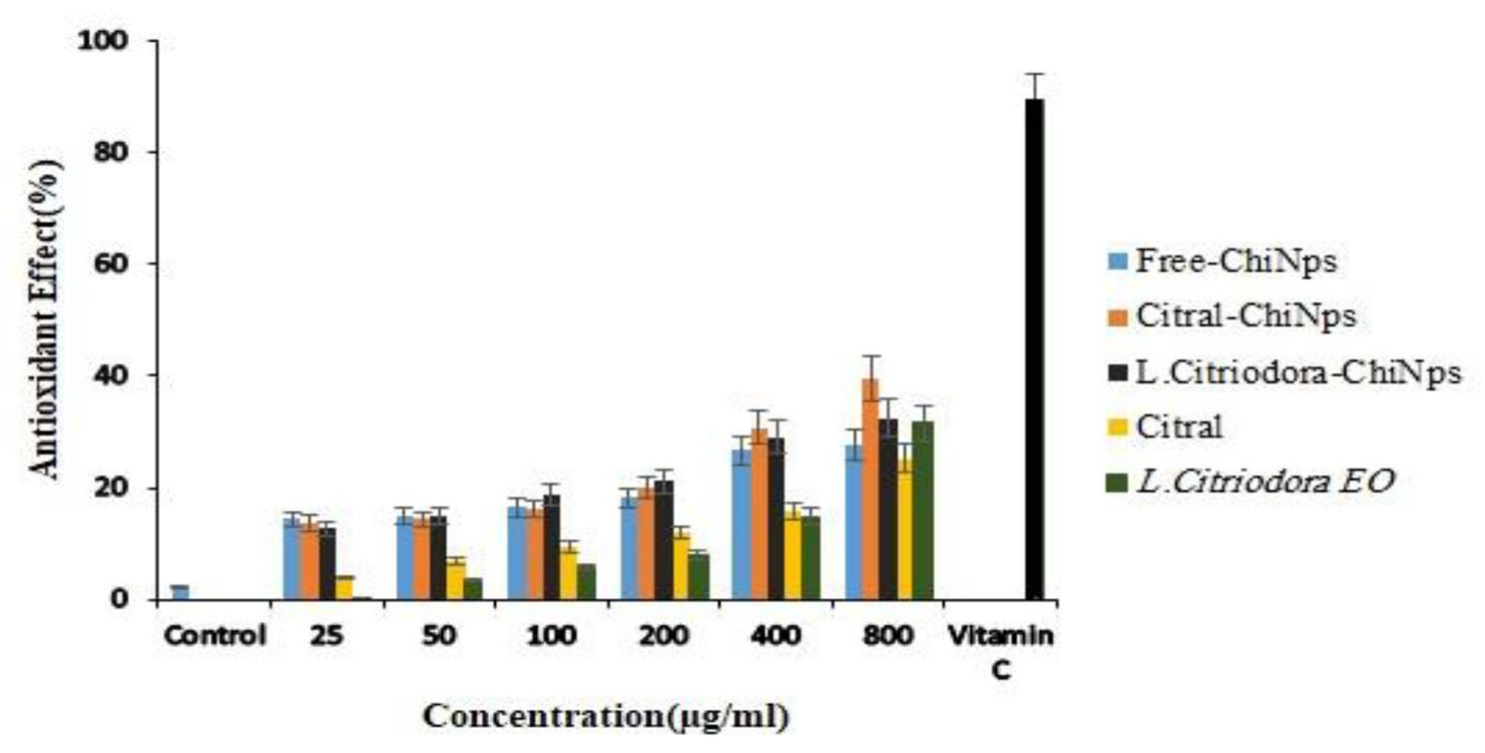
Antioxidant effects of Free-ChiNPs, Citral-ChiNPs, L. citriodora-ChiNPs, citral, and *L. citriodora* EO at different concentrations

Figure 4 shows the antioxidant activity of Free-ChiNPs, Citral-ChiNPs, L. citriodora-ChiNPs, citral, and *L. citriodora* EO at 25–800 µg/mL concentrations. Dose-response effects are observed between the concentration of the samples and their antioxidant activity. Citral-ChiNPs with approximately 40% activity at 800 µg/mL showed the best efficacy.

### The cytotoxicity effects of samples

**Fig. 5:**
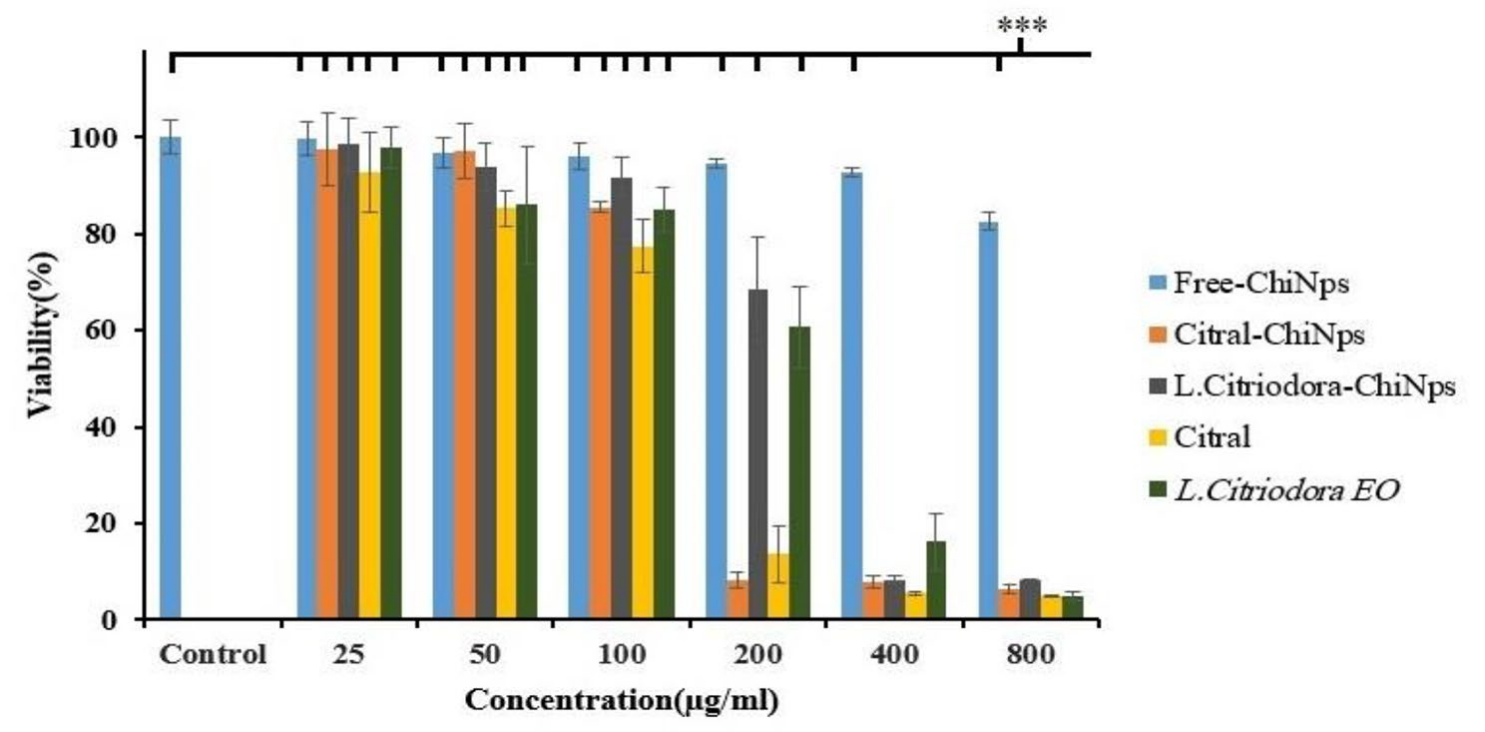
Cytotoxicity effects of Free-ChiNPs, Citral-ChiNPs, L. citriodora-ChiNPs, citral, and *L. citriodora* EO on A375 cells. Viability(%) of treatment groups in different concentration in comparison to Citral-ChiNPs(800 µg/ml) ***p value < 0.001

Figure 5 displays the effect of various concentrations of Free-ChiNPs, Citral-ChiNPs, L. citriodora-ChiNPs, citral, and *L. citriodora* EO on A375 cell viability. Free-ChiNPs did not significantly affect cell viability (< 10%). After treatment with Citral-ChiNPs, L. citriodora-ChiNPs, citral, and *L. citriodora* EO, the cell viability was decreased in a dose-dependent manner. The IC50 values of citral and *L. citriodora* EO were 136.9 µg/ml and 240.1 µg/ml, respectively, and there was no significant difference between them (*p* value = 0.137). Moreover, the IC50 values of Citral-ChiNPs and L. citriodora-ChiNPs were 105.6 µg/ml and 199.9 µg/ml, respectively (*p* value < 0.05).

### Effect of the samples on the expression of apoptosis regulatory genes (Bax and Bcl-2)

**Fig. 6:**
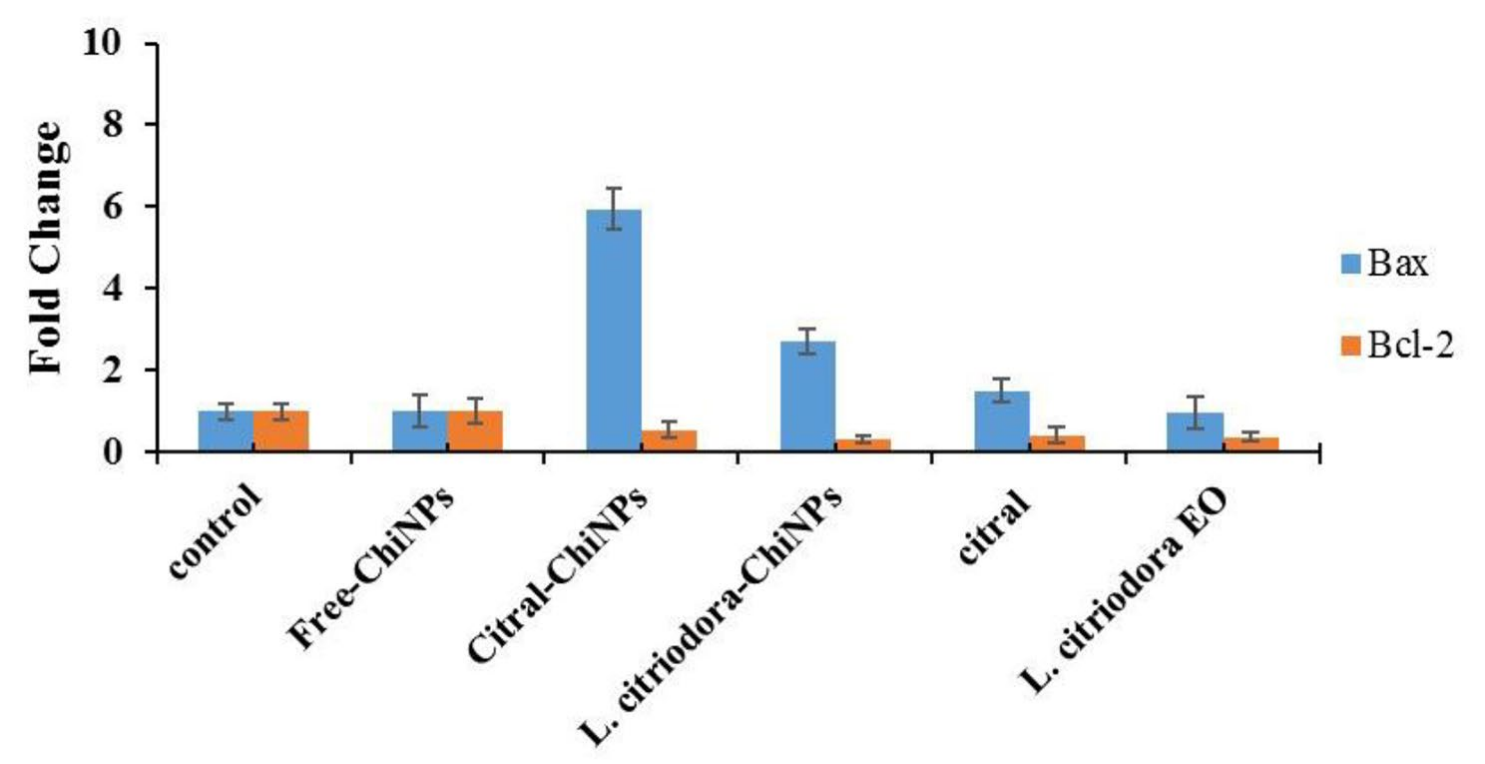
Bax and Bcl-2 gene expression in the A375 cell line after treatment with Free-ChiNPs, Citral-ChiNPs, L. citriodora-ChiNPs, citral, and *L. citriodora* EO. BAX/BCL-2 ratio of treatment groups in comparison to Citral-ChiNPs *P Value < 0.05, **P Value < 0.01, ***P Value < 0.001

Figure 6 presents the expression of the Bax and Bcl-2 genes. The ratio of Bax/Bcl2 in the Free-ChiNPs group was approximately equal to that in the control group (= 1). In addition, the Bax/Bcl-2 ratio fold change in the treated group with Citral-ChiNPs was 11.18, L. citriodora-ChiNPs 9.06, citral 3.75, and *L. citriodora* EO 2.69. Considering that the ratio of the expression of the apoptosis gene (Bax) to the inhibitory gene (Bcl-2) was higher than 1 in all the samples, the conditions for the apoptosis of the cells after treatment with them are ready.

### Apoptosis detected by flow cytometry

**Fig. 7:**
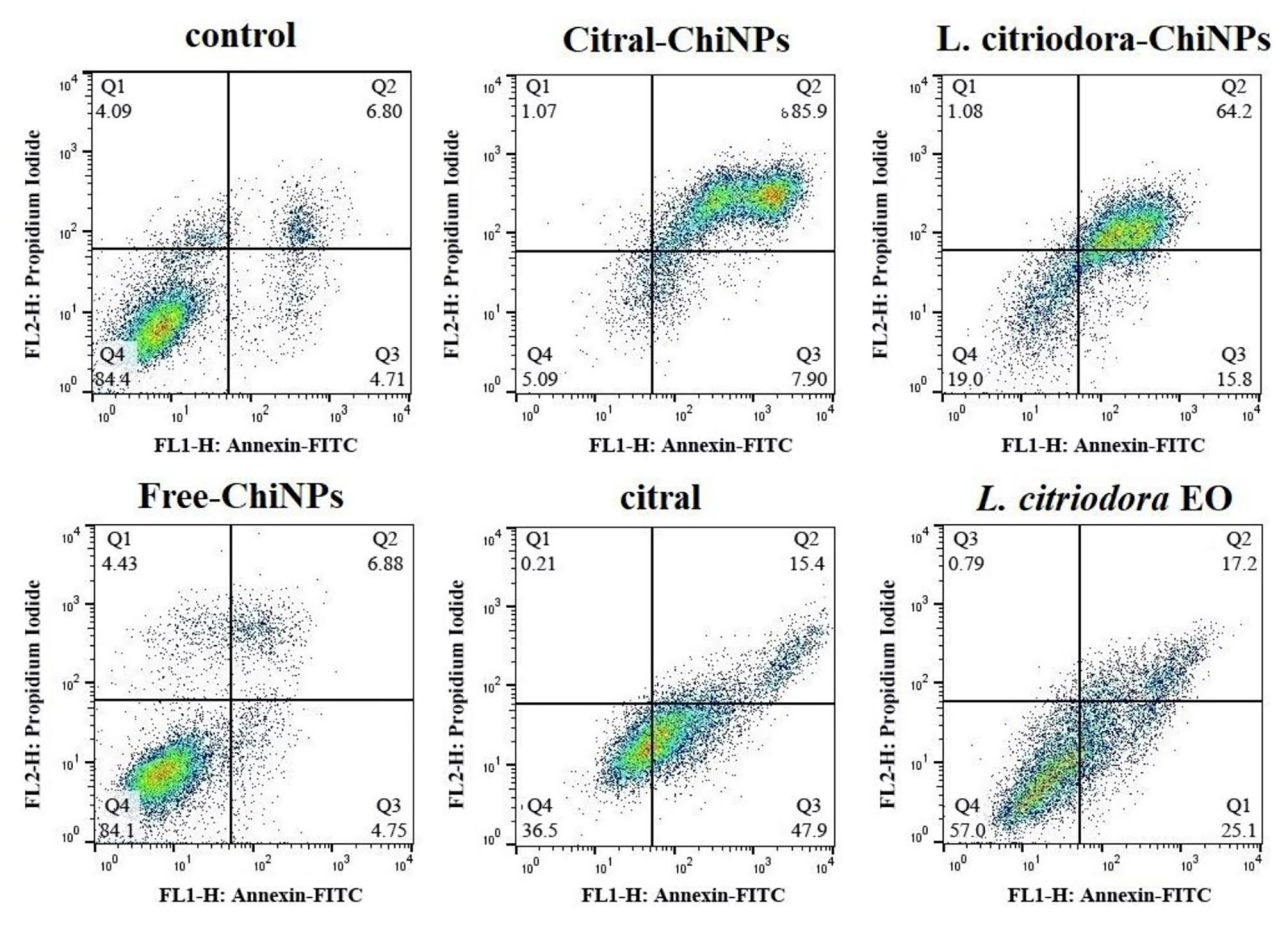
Flow cytometry analysis in A375 cells treated (24 h) with Free-ChiNPs, Citral-ChiNPs, L. citriodora-ChiNPs, citral, and *L. citriodora* EO. The histogram shows 11 ± 0.5% of cells at total (early & late) apoptotic (Annexin V + PI+) in control, 12 ± 0.5% in Free-ChiNPs, 93.5 ± 0.3% in Citral-ChiNPs, 80 ± 0.2% in L. Citriodora-ChiNPs, 63 ± 0.3% in Citral and 42 ± 0.3 in *L. citriodora* EO treated cells

The distribution of viable (Annexin V - PI-), necrotic (Annexin V- PI+), early apoptotic (Annexin V + PI-), and late apoptotic (Annexin V + PI+) cells is presented in Fig. 7. Compared to the control group (4.71 and 6.8%), the citral-ChiNPs induced early apoptosis by 7.9% and late apoptosis by 85.9%. Additionally, the percentages of early and late apoptosis induced by L. citriodora-ChiNPs (15.8–64.2%), citral (47.9–15.4%), *L. citriodora* EO (25.1–17.2%), and Free-ChiNPs (4.75–6.68%) were compared to the control group.

## Discussion

In this study, *L. citriodora* EO was used as a natural anticancer agent. Citral (25.9%) was identified as its main constituent. The results of other studies also show that citral is the main ingredient of *L*. *citriodora* EO [27, 28]. Citral (geranial) is a noncyclic monoterpene aldehyde (C10H16O) insoluble in water with anticancer, antioxidant, antimicrobial, anti-inflammatory, and antidiabetic properties [29, 30].

The current study used the ionic gelation method to prepare nanoparticles containing *L. citriodora* EO and citral. In other studies, *L. citriodora* EO and citral nanoemulsions have been reported; however, their chitosan nanoparticles were not reported [31, 32]. This study explored the potential anticancer effects of *L. citriodora* EO and its major compound, citral, on the A375 melanoma cell line. To enhance their cytotoxicity against A375 cells, chitosan nanoparticles containing EO and citral were prepared. The results showed that treatment with 200 and 100 µg/ml of L. citriodora-ChiNPs and Citral-ChiNPs decreased cell viability by 25% and 28%, respectively. Some reports on proposing chitosan nanoparticles containing EOs have been published. For example, viability of A375 cells after treatment with 75 µg/mL *Syzygium aromaticum* EO and chitosan nanoparticles containing this EO were observed at 97 and 45% [33]. Another study showed chitosan nanoparticles loaded with *Morinda citrifolia* EO (40 µg/ml) have 54% cell cytotoxicity on A549 human lung cancer cells [34]. In another study, researchers reported that chitosan nanoparticles loaded with celandine (*Chelidonium majus* L.) EO had more significant cytotoxicity on the MCF-7 cell line compared to its non-nano form; cell viability was 32 and 63%, respectively [35].

This study investigated the antioxidant and anti-cancer activity of EO and citral and their nanoparticle forms. The antioxidant power increased with the increase in concentration, which has been confirmed in other studies [36, 37]. The human body is affected by a variety of disorders that have been linked to free radicals. Free radicals can cause damage to proteins, DNA, and RNA, leading to genetic mutations that can contribute to cancer development. All living cells express an antioxidant defence system to combat the harmful effects of free radicals. This system can be classified in various ways depending on the antioxidant source. Antioxidants can be obtained from dietary intake (exogenous) or produced by the body (endogenous). Research has demonstrated that antioxidants reduce cancer incidence [38, 39].

Cancer cells can survive by upregulating the anti-apoptotic BCL2 protein and downregulating the pro-apoptotic Bax protein. Modifying the ratio of Bax to BCL2 and increasing the level of Bax causes the accumulation of Bax in the endoplasmic reticulum and mitochondrial membranes, which facilitates the release of apoptogenic proteins such as cytochrome c and then caspase, which subsequently leads to apoptosis [40, 41]. EOs have been found to trigger programmed cell death in cancer cells through various mechanisms, including apoptosis, necrosis, cell cycle arrest, and disruption of vital cellular organelles [42, 43]. In the current investigation, a notable difference was observed in the expression of these two genes in cells treated with citral-ChiNPs and L. citriodora-ChiNPs compared to the citral, EO, and control groups.

In the present study, flow cytometry was applied to measure the amount of apoptosis and distinguish it from necrosis in cells exposed to environmental stressors and decomposition. The findings showed that Citral-ChiNPs resulted in the highest percentage of cell death through apoptosis. Based on the findings from flow cytometry and qPCR analysis, it can be inferred that the cells underwent programmed cell death, leading to alterations in the expression of apoptotic genes.

Numerous mechanisms have been proposed for the mode of action of citral that induces apoptosis in cancer cells. One of these mechanisms is the intrinsic or mitochondrial pathway, initiated by releasing cytochrome c from the mitochondria, activating a cascade of caspases that ultimately leads to apoptosis [30]. Another mechanism involves the inhibition of proteins that are involved in cancer cell survival and proliferation. For instance, one study showed that citral inhibited the activity of the protein STAT3 in small-cell lung cancer, leading to decreased cell viability and increased apoptosis [44].

## Conclusion

The chemical composition of *L. citriodora* EO was investigated, and citral was identified as the major compound. Then, a comprehensive comparison was made between the antioxidant and cytotoxic effects of the EO and citral and chitosan nanoparticles containing them against A375 cells, with higher anticancer effects and antioxidant properties. The cytotoxic effect of chitosan nanoparticles containing citral (Citral-ChiNPs) was more potent than that of the other samples, and it also had a higher antioxidant effect.

## Data Availability

All data are available from the corresponding authors upon reasonable request.

## List of abbreviations

GC–MS: Chromatography-Mass Spectrometry
EO: Essential Oil
ATR-FTIR: Attenuated Total Reflection-Fourier Transform InfraRed
TEM: Transmission Electron Microscope
ChiNPs: Chitosan nanoparticles
Citral-ChiNPs: chitosan nanoparticles containing citral
L. citriodora-ChiNPs: chitosan nanoparticles containing L. citriodora essential oil
